# Supplementary material for: COZOID: contact zone identifier for visual analysis of protein-protein interactions
Source: BMC Bioinformatics. 2018 Apr 6;19:125. doi: 10.1186/s12859-018-2113-6 (PMC5889581; doi:10.1186/s12859-018-2113-6)
Supplement: Supplementary file 2 — Software build. Executable binary file of the software tool. (ZIP 118,784 kb) [file 12859_2018_2113_MOESM2_ESM.zip › cozoid/java/docs/beansbinding-1.2.1-doc.zip!/overview-frame.html]

Overview List (Beans Binding)


All Classes

Packages
  
org.jdesktop.beansbinding
  
org.jdesktop.beansbinding.ext
  
org.jdesktop.el
  
org.jdesktop.el.impl
  
org.jdesktop.el.impl.lang
  
org.jdesktop.el.impl.parser
  
org.jdesktop.el.impl.util
  
org.jdesktop.observablecollections
  
org.jdesktop.swingbinding
  
org.jdesktop.swingbinding.adapters
  
org.jdesktop.swingbinding.impl
